# Global Vectors Representation of Protein Sequences and Its Application for Predicting Self-Interacting Proteins with Multi-Grained Cascade Forest Model

**DOI:** 10.3390/genes10110924

**Published:** 2019-11-12

**Authors:** Zhan-Heng Chen, Zhu-Hong You, Wen-Bo Zhang, Yan-Bin Wang, Li Cheng, Daniyal Alghazzawi

**Affiliations:** 1 The Xinjiang Technical Institute of Physics and Chemistry, Chinese Academy of Sciences, Urumqi 830011, China; chenzhanheng17@mails.ucas.ac.cn (Z.-H.C.); zhang_wen_bo@foxmail.com (W.-B.Z.); wangyanbin15@mails.ucas.ac.cn (Y.-B.W.); chengli@ms.xjb.ac.cn (L.C.); 2 University of Chinese Academy of Sciences, Beijing 100049, China; 3 Department of Information Systems, King Abdulaziz University, Jeddah 21589, Saudi Arabia; dghazzawi@kau.edu.sa

**Keywords:** self-interacting proteins, de novo protein sequence, global vector representation, multi-grained cascade forest

## Abstract

Self-interacting proteins (SIPs) is of paramount importance in current molecular biology. There have been developed a number of traditional biological experiment methods for predicting SIPs in the past few years. However, these methods are costly, time-consuming and inefficient, and often limit their usage for predicting SIPs. Therefore, the development of computational method emerges at the times require. In this paper, we for the first time proposed a novel deep learning model which combined natural language processing (NLP) method for potential SIPs prediction from the protein sequence information. More specifically, the protein sequence is de novo assembled by *k-mers*. Then, we obtained the global vectors representation for each protein sequences by using natural language processing (NLP) technique. Finally, based on the knowledge of known self-interacting and non-interacting proteins, a multi-grained cascade forest model is trained to predict SIPs. Comprehensive experiments were performed on *yeast* and *human* datasets, which obtained an accuracy rate of 91.45% and 93.12%, respectively. From our evaluations, the experimental results show that the use of amino acid semantics information is very helpful for addressing the problem of sequences containing both self-interacting and non-interacting pairs of proteins. This work would have potential applications for various biological classification problems.

## 1. Introduction

Proteins perform a vast array of functions within organisms. Their self-interaction needs to be considered for the full understanding of cell functions and biological phenomena. However, it is always an important task to identify the interaction between proteins because of the large data it contains in the post-genome era. The prediction of self-interacting proteins (SIPs) will offer a wide understanding to drug target detection [1], drug discovery [2,3], and even further biological processes [4]. According to investigation, the previous biological experimental studies [5,6] have many disadvantages such as high cost, time-consuming, low efficiency and so on. In order to efficiently predict SIPs, many researchers try their best to draw attention to develop new strategies.

From the past years, several researchers have implemented a tremendous work to generate the protein–protein interactions (PPIs) data, which will provide help for discovering the SIPs. Salwinski et al. established easily accessible online database of interacting proteins, which can be utilized to identify the most reliable subset of the interactions [7]. Chart-Aryamontri et al. updated the biological general repository for interaction datasets that stored the important information of protein, genetic and chemical interactions for humans and organism species [8]. Szklarczyk et al. collected and integrated the functional interactions between expressed proteins, and constructed the STRING database by consolidating known PPIs data [9]. Based on these widely known PPIs datasets, Liu et al. integrated and built *human* and *yeast* datasets for SIPs detection [10].

Currently, a large scale of methods have been exploited to predict PPIs [11,12,13,14]. Jansen et al. developed an approach applying Bayesian networks to predict PPIs from genomic data, which can naturally weight and combine into reliable predictions genomic features only weakly associated with interaction [15]. Ofran and Rost predict directly from the sequence of a single protein which residues are interaction hotspots without known of their partner, this research makes it possible to annotate and analyze the hotspots of PPIs in the whole organism, which is conducive to functional prediction and drug development [16]. Zhang et al. combined three-dimensional structural information with other functional clues to predict PPIs on a genome-wide scale, which was comparable in accuracy to high-throughput experiments [12]. Sun et al. studied the sequence-based PPI prediction by applying a stacked autoencoder method, which was the first to use deep-learning algorithm to sequenced-based PPI prediction [17]. Kovács et al. studied PPIs prediction methods on the basis of biological or network-based similarity, and discovered that proteins interact not if they are similar to each other, but if one of them is similar to the other’s partners [18]. However, these approaches could be applied to detect PPIs well [19,20], but they are not good enough to predict SIPs. They mainly exist in the following points: (1) In essence, they also have certain limitations that take the correlation between protein pairs into account for SIPs detection, for example co-expression, co-localization, and co-evolution. Nevertheless, this information has no use for SIPs. (2) In addition, the datasets applied to predict PPIs are different from those of SIPs, the datasets of the former are balanced and those of the latter are unbalanced. (3) Besides, there is no PPIs between same partners in the datasets. In virtue of reasons, these computational methods are not suitable for detecting SIPs.

Recently, the researchers found that some similarities between human language and biological language. Nevertheless, it is quite difficult for people to discover the true meaning of biological patterns different from human language. Some researchers have attempted to introduce natural language processing (NLP) technology to the field of bioinformatics. Anon George et al. used NLP technique to extract features and give appropriate representation for the protein sequences, which can better understand the semantics of protein sequences [21]. Wang et al. developed a biological language processing model called bio-to-vector (Bio2Vec) for PPIs detection based on convolution neural network (CNN) [22]. Wan et al. proposed a new scheme for predicting compound–protein interactions by combining feature embedding with deep learning, which used several NLP techniques to extract important features from proteins and compounds [23]. However, seldom do researchers introduce NLP technique to predict SIPs.

In our study, inspired by recent work in NLP technique and deep learning [24,25,26], we put forward a multi-grained cascade model for SIPs prediction base on global vectors representation of de novo protein sequence. Furthermore, the major advantages of our method include the following three aspects: (1) *k-mers* method was exploited to de novo assemble protein sequence; (2) we employed global vectors (GloVe) representation learning method to generate feature vector of each *mer* from de novo protein sequence, a 100-Dimensional feature vector from the numerical series was achieved by this method; and (3) multi-grained cascade model was applied to optimize the characteristics and predict SIPs. In detail, we first used *3-mers* to de novo assemble every protein sequence from the corresponding datasets, and each protein sequence was regarded as a “sentence”, every *mer* in a “sentence” was treated as a “word”. Then, GloVe model was applied for emerging 100-dimensional feature vectors of each *mer*, which can contain, as much as possible, the semantic information between *mers*. Finally, the feature vectors of all protein sequences were fed into a multi-grained cascade forest classifier to predict SIPs. We have tested our model on *yeast* and *human* datasets. The experimental results demonstrated that the superior performance of our model than the other previous methods in predicting new SIPs. It is revealed that the presented method is suitable and perform well for detecting SIPs.

## 2. Materials and Methods

### 2.1. Benchmark Datasets Preparation

As we all know that the PPIs related information can be achieved from many different types of resources, including DIP [7], InnateDB [27], IntAct [28], BioGRID [8] and MatrixDB [29]. In this study, the datasets constructed in the experiment which contains 20,199 curated *human* protein sequences mainly derived from the UniProt database [30]. We mainly set up the SIPs datasets for the experiment which embodies two identical interacting protein sequences and whose type of interaction was characterized as “direct interaction” in relational databases. On this foundation, we can obtain 2994 *human* self-interacting protein sequences which would be employed to construct the datasets for the experiment.

We need to select the datasets from the 2994 *human* SIPs for the experiment to measure the performance of our prediction model, which mainly includes three steps [10]: (1) We removed the protein sequences which may be fragments, and retained the length of protein sequences more than 50 residues and less than 5000 residues from all the *human* proteome; (2) to build the *human* positive dataset, we chose a high quality SIPs data which should conform to one of the following conditions: (a) The self-interactions were revealed by at least one small-scale experiment or two sorts of large-scale experiments; (b) the protein has been announced as homo-oligomer (containing homodimer and homotrimer) in UniProt; (c) it has been reported by more than two publications for the self-interactions; and (3) for the *human* negative dataset, we removed all the types of SIPs from the whole *human* proteome (including proteins annotated as ‘direct interaction’ and more extensive ‘physical association’) and SIPs detection in UniProt database. Eventually, the ultimate *human* dataset for the experiment was consisted of 1441 SIPs and 15,938 non-SIPs [10].

In the same way, we also generated the *yeast* dataset to further measure the cross-species capacity of our multi-grained cascade forest model by repeating the same strategy mentioned above. Finally, the *yeast* benchmark dataset was consisted of 710 positive sample and 5511 negative sample [10].

### 2.2. De Novo Assembly Protein Sequences

**Figure 1:**
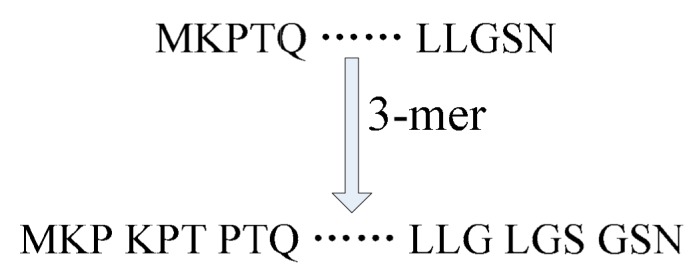
De novo assembled protein sequences by *3-mer*.

Actually, there is a close relation between biological language and human natural language. De novo assembly protein sequence is a helpful tool for understanding the relationship between amino acids in biological language. The protein sequences could be regarded as sentences, and the monomeric units (*mer*) were treated as words. The *k-mer* is a de novo protein sequence assembly method in the present study, which could be employed to; divide a sequence into many sets of *k* amino acid residues. In the Figure 1, the protein sequences were de novo assembled by *3-mers* composition [31]. A series of new amino acid compositions were created for each protein sequence [32]. For example, there is one protein sequence with length *m*, and every *3-mers* composition amino acids were regarded as a “word”. Then, the protein sequence will be de novo assembled by m-2 *3-mers*.

In conclusion, *k-mer* has become essential to de novo assembly protein sequences for predicting self-interacting proteins. Each protein sequence was de novo assembled by *k-mer* algorithm. Then, these entire *mers* were saved into a text file, which can be employed for building co-occurrence matrix and obtaining the feature vectors.

### 2.3. Global Vectors Representation of Protein Sequences

In natural language processing (NLP), global vectors representation (i.e., GloVe) is an ideal tool for obtaining vectors corresponding to each word in a corpus [33]. GloVe used global and local statistical information of words to generate language model and word vectors. At present, there are two main types of vector representations: (1) Global matrix factorization methods, such as latent semantic analysis (LSA) [34]; (2) local context window methods, such as skip-gram model [35]; GloVe combines the advantages of these two methods, it can not only accelerate the training speed of the model, but also control the relative weight of words. This model consists of two steps: Construction of co-occurrence matrix and generation of global vectors.

The statistics of *mer–mer* co-occurrences in a protein sequence is the key source of information. First of all, let the co-occurrence matrix be *X*, where *X_i,j_* represents the frequency of *i* and *j* appear together in a window (the size of window is 7). There was one sentence with several words as follow:

MKP KPT PTQ TQD QDS DSQ SQE QEK EKV ……LLG LGS GSN

Supposing that the central *mer* is TQD, while the contextual *mers* are MKP, KPT, PTQ, QDS, DSQ, and SQE. The number of *mer–mer* co-occurrences is calculated as follows:

(1) $$X_{TQD,MKP}+=1$$

(2) $$X_{TQD,KPT}+=1$$

(3) $$X_{TQD,PTQ}+=1$$

(4) $$X_{TQD,QDS}+=1$$

(5) $$X_{TQD,DSQ}+=1$$

(6) $$X_{TQD,SQE}+=1$$

The co-occurrence matrix *X* will be obtained by traversing the whole sequences through a sliding window.

Next, we constructed the approximate relationship between word vector and the co-occurrence matrix as follow:

(7) $$v_{i}^{T}v_{j}+b_{i}+b_{j}=log(X_{ij})$$

where *v_i_*, *v_j_* are the vectors of *mer i* and *j* respectively; *b_i_*, *b_j_* are two additional scalars (biases). Then, the cost model will be built as Formula (8) [33].

(8) $$J=\sum_{i,j}^{N}f(X_{ij}){\left(v_{i}^{T}v_{j}+b_{i}+b_{j}-\log(X_{ij})\right)}^{2}$$

where, *N* is the size of dataset, and the size of co-occurrence matrix is N*N; *f*(*X_ij_*) is the weighting function which should obey the following condition:

(9) $$f(x)=\left\{\begin{array}{cc} {\left(x/x_{max}\right)}^{\alpha } & if\;x<x_{max}\\ 1 & otherwise \end{array}\right.$$

In our research, while training the model, most hyper-parameters were set by default, but a few parameters still need to be set. The gradient descent algorithm based on AdaGrad was used to randomly sample all non-zero elements of matrix *X*. We also set *learn_rate* = 0.05, *vector_size* = 100, *iterations* = 25, *window_size* = 7 and *min_count* = 0. Hence, each protein sequence of the corresponding dataset can be obtained a 100-dimensional vector by applying GloVe model.

### 2.4. Multi-Grained Cascade Forest

In 2017, Zhou et al. proposed a novel decision tree ensemble method called multi-grained cascade forest [36,37], with less hyper-parameters than deep neural networks (DNN) [38,39]. There are two stages in the training process of multi-grained cascade forest model: Multi-grained scanning and cascade forest. The multi-grained scanning was used to generate feature values, cascade forest was applied to obtain the prediction results through multiple forest cascades.

#### 2.4.1. Multi-Grained Scanning

**Figure 2:**
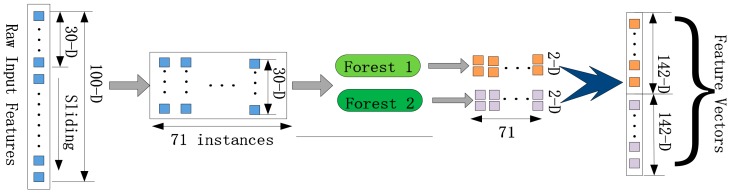
Process of multi-grained scanning.

In our model, in order to improve feature representation, we used sliding window to scan the raw feature vectors and input them into forest to generate new features. Thereby, the cascade forest could be enhanced by the multi-grained scanning method. The scanning process is shown Figure 2.

There are 2 classes in our experiment, and the raw feature vectors were obtained by GloVe method, whose size was 100. We process these feature vectors by using a 30-dimensional sliding window. And then, in total 71 instances were produced. These instances extracted from the same windows were employed to train a random forest and a completely-random tree forest. Subsequently, each forest will achieve 142-dimensional derived vectors. As shown in Figure 2, the final class vectors were obtained and concatenated by these derived vectors, which will be input into the cascade forest.

#### 2.4.2. Cascade Forest

**Figure 3:**
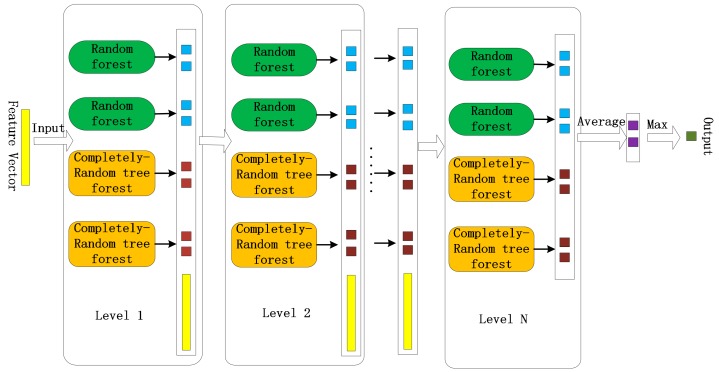
Cascade forest model.

On the basis of representation learning in DNN, we employed cascade forest to predict the SIPs based on layer-by-layer processing of input feature vectors. The main thought is to conjecture better results through multilayer cascade forest. This method also called ensemble of ensembles. Every layer of cascade forest used a set of forests to simulate the representation, and each forest was consisted of many decision trees, and each layer of forest applied the information of the upper layer as its own input, while its output provides input information for the next layer. The cascade forest model is shown in Figure 3.

As is shown in Figure 3, every layer of cascade forest was composed of 2 random forests and 2 completely-random tree forests. The number of trees in these forests were set with default parameters. For the completely-random tree forests, each node of every decision tree randomly chose one feature to split until all the instances of each leaf node belongs to the same class or the number of instances is less than 10. For the random forests, each decision tree was generated by randomly selecting *sqrt(d)* features (*d* is the total input features), and then chose the maximum feature of *gini* coefficient as the condition of node partition. In the experiment, after layer-by-layer processing of input feature vectors, each forest will generate a 2-dimensional predictive probability distribution vector. Then, to average the four 2-dimensional vectors at the last level. Finally, we can achieve the final prediction results with the maximum aggregated value.

### 2.5. Performance Evaluation Indicators

As to classification model, there are 3 main evaluation indicators: Confusion matrix (CM), receiver operating characteristic (ROC) curve and area under ROC curve (AUC). CM is an important performance assessment tool for our proposed classification model. We can establish a table contained four underlying indexes as follow:

From the Table 1, each row of the matrix is the situation of true samples and each column of matrix represents the situation of predicted samples. Meaning of the four underlying indexed (also called primary indices) are as follow:

(1) TN: True negative, the number of true non-interacting pairs correctly predicted;
(2) FN: False negative, the quantity of true non-interacting pairs falsely predicted;
(3) FP: False positive, the count of true interacting pairs falsely predicted;
(4) TP: True positive, the quantity of true interacting pairs correctly predicted.

However, in the face of a large number of data, CM is hard to be used to measure the quality of the model only by the number of statistic samples. On the basis of those parameters of CM, we calculated the four values which called secondary indicator as follow:

(10) $$Acc=\frac{TP+TN}{TP+FP+TN+FN}$$

(11) $$TNR=\frac{TN}{FP+TN}$$

(12) $$F1-score=\frac{2TP}{2TP+FP+FN}$$

(13) $$MCC=\frac{\left(TP\times TN)-(FP\times FN\right)}{\sqrt{\left(TP+FN)\times (TN+FP)\times (TP+FP)\times (TN+FN\right)}}$$

where,

(1) Acc: Accuracy, the proportion of all judged correctly samples in the total observation values from the classification model;
(2) TNR: True negative rate or specificity, the proportion of predicted correctly samples in the result with actual negative value;
(3) F1-score: Measuring the overall performance of the classification model;
(4) MCC: Matthews correlation coefficient, the geometric mean of the problem and dual regression coefficients; It is a better indicator for measuring unbalanced dataset and the most informative single fraction for assessing the quality of binary classifier from the CM.

Moreover, a receiver operating curve (ROC) was plotted to evaluate the performance of random projection method. And then, we can compute the area under curve (AUC) to evaluate the quality of the classifier.

## 3. Results and Discussion

### 3.1. Performance Evaluation on Protein Self-Interaction

We first assessed the proposed method on the SIPs extracted from *yeast* dataset. In order to avoid the risk of over-fitting, *k*-fold cross validation was applied to the class vectors generated by each forest in our model. More concretely, we only separated the datasets which were mainly composed of characteristic values into *k* non-overlapping pieces, and each training sample was used *k* − 1 times in forest to generate *k* − 1 class categories list, and then averaged them to generate the final result as the enhancement feature of the next level in the cascade forest. Because cross validation has been done in the process of multi-grained cascade forest model building, it is not necessary to introduce it again when using our proposed model to realize classification prediction. To illustrate the rationality, toughness and stability of our algorithm, we also implemented the method of multi-grained cascade forest on the *human* dataset.

To guarantee impartiality and objectivity of the test, the parameters for *human* and *yeast* datasets should be set in the same way. In our task, compared with DNN, multi-grained cascade forest model has fewer hyper-parameters and much easier to train. When processing various of data in different fields, it can achieve excellent performance under almost identical hyper-parameter settings, in other words, it has high robustness for our model to set hyper-parameters. Because the model is insensitive to the process parameter change, and a set of hyper-parameters can be applied to different datasets. Hence, apart from a few parameters, most of them were set by default. In the experiment, we set *shape_1X* = 30 (shape of a single sample unit. Required when using multi-grained scanning), *window* = 30 (the size of sliding window during multi-grained scanning), *tolerance* = 5.0 (accuracy tolerance for the cascade growth). If the improvement in accuracy is not better than the tolerance, the construction is stopped.

Afterwards, we test our prediction model on *yeast* and *human* benchmark datasets, and the results are shown in Table 2. From the data, it is revealed that our proposed model exhibited the outcomes of Acc, TNR (Specificity), F1-score and MCC of 91.45%, 99.71%, 37.56%, and 0.4389 respectively on *yeast* dataset. Similarly, we can obtain the results by running experiment on *human* dataset, the Acc is 93.12%, TNP is 99.57%, F1-score is 39.10%, and MCC is 0.4421.

Meanwhile, receiver operating characteristic (ROC) curves was widely applied in many fields, such as machine learning, data mining, and so on. We also used ROC curves to measure the comprehensive index between false positive rate and true positive rate continuous variable. The area under curves (AUC) could be shown the prediction accuracy of the classifier. The larger the AUC, the higher the accuracy.

**Figure 4:**
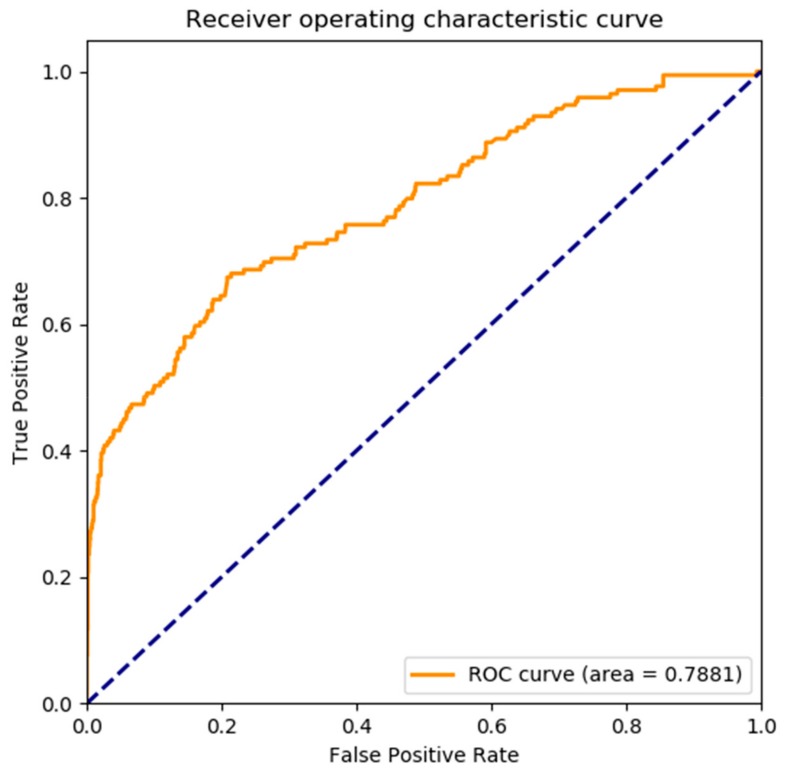
The receiver operating characteristic (ROC) curve of proposed model on *yeast* dataset.

**Figure 5:**
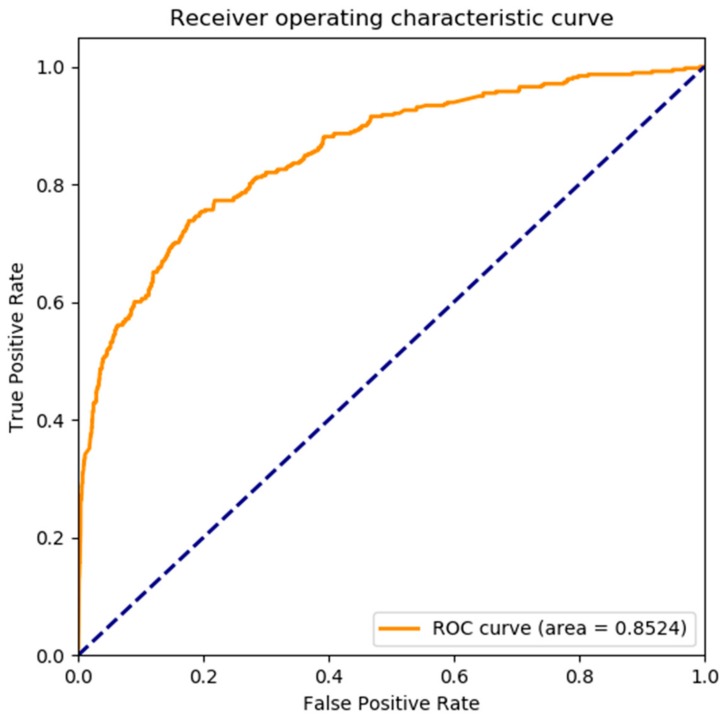
The ROC curve of proposed model on *human* dataset.

The ROC curve of our proposed model on *yeast* dataset is shown in Figure 4, it is obvious that the AUC is 0.7881. The ROC curve of the proposed model on *human* dataset is shown in Figure 5, it is clear that the AUC is 0.8524. That is to say, our model performs better on large scale dataset than the small dataset. Overall, the presented model is an accurate and robust classifier for predicting SIPs.

### 3.2. Comparison with Other Existing Methods for Predicting SIPs

To further measure the quality of our proposed model, we compared the proposed model with other previous methods based on the two benchmark datasets. The comparison results were listed a clear statement of account in Table 3 and Table 4. From Table 3, it is obvious that the multi-grained cascade forest model obtained the highest accuracy of 91.45% than the other six methods (range from 66.28% to 87.46%) on *yeast* dataset. At the same instant, it is clear to see that the other six methods got lower MCC (range from 0.1577 to 0.2842) than our proposed model of 0.4389 on the same dataset. In exactly the same way, from Table 4, the overall results of our prediction approach is also significantly better than the other six methods on *human* dataset. To make a summary, we assessed our multi-grained cascade forest model against with the other six approaches on both *yeast* and *human* datasets, so that the prediction accuracy of the overall experimental results could be improved. This fully illustrates that a reasonable feature representation method and a suitable classifier are very significant for predicting SIPs. It is further illustrated that the proposed method is superior to the other six approaches and quite suitable for predicting SIPs.

As mentioned above, it is apparent that our method can receive good effect of SIPs detection because of the appropriate feature representation and classifier. The presented feature representation technique plays a critical part in enhancing the prediction accuracy. The specific reasons can be summed up in the following three aspects: (1) *k-mer* method was exploited to de novo assemble protein sequence. Not only can it represents the information of protein sequence, but also it preserves useful enough information as much as possible; (2) we employed global vectors (GloVe) representation learning method to generate feature vector of each *mer* from de novo protein sequence, a 100-Dimensional feature vector from the numerical series was achieved by this method. Hence, the protein sequences can be described in the form of numerical values; and (3) multi-grained cascade forest model was applied to optimize the characteristics and predict SIPs. In a few words, experimental results revealed that our presented model is extreme fit for SIPs prediction.

## 4. Conclusions

In this study, a multi-grained cascade forest-based model was developed for predicting SIPs based on protein primary sequence. To better understand the aggregation relationship among amino acids and discover the semantic information of proteins, we proposed an improved global vectors representation learning scheme from the de novo assembled protein sequence based on natural language processing technique. We implement our model on *yeast* and *human* SIPs datasets, each protein sequence can be de novo assembled by *3-mers* technique and obtained a 100-dimensional feature vector. Afterwards, we evaluated the performance of our proposed model on the two benchmark datasets and also compared with other popular methods, which achieved an accuracy rate of 91.45% and 93.12% respectively. Experimental results revealed that our method has better performance than other existing approaches. We conjecture that de novo assembly protein sequence combined with GloVe representation may play an important role in the SIPs prediction and help to increase efforts in discovering amino acid words. For the future work, there will be more effective NLP methods and deep learning techniques introduced for detecting SIPs.

## 5. Patents

This work has been applied for the national invention patent of China.

## Figures and Tables

**Table 1:** Confusion matrix. TN: true negative, FN: false negative, FP: false positive, TP: true positive.

		Predict	
		Negative	Positive
Actual	Negative	TN	FN
	Positive	FP	TP

**Table 2:** Performance of our proposed model on the two benchmark datasets. Acc: Accuracy; TNR: True negative rate; F1-score: Measuring the overall performance of the classification model; MCC: Matthews correlation.

Datasets	Acc (%)	TNR (%)	F1-Score (%)	MCC
***yeast***	91.45	99.71	37.56	0.4389
***human***	93.12	99.57	39.10	0.4421

**Table 3:** Performance of our proposed model and other previous methods on *yeast* dataset. AUC: Area under curve.

Model	Acc (%)	TNR (%)	F1-Score (%)	MCC	AUC
SLIPPER [40]	71.90	72.18	36.16	0.2842	0.7723
DXECPPI [41]	87.46	94.93	34.89	0.2825	0.6934
PPIevo [42]	66.28	87.46	28.92	0.1801	0.6728
LocFuse [43]	66.66	68.10	27.53	0.1577	0.7087
CRS [10]	72.69	74.37	33.05	0.2368	0.7115
SPAR [10]	76.96	80.02	34.54	0.2484	0.7455
**Proposed method**	**91.45**	**99.71**	**37.56**	**0.4389**	**0.7881**

**Table 4:** Performance of our proposed model and other previous methods on *human* dataset.

Model	Acc (%)	TNR (%)	F1-score (%)	MCC	AUC
SLIPPER [40]	91.10	95.06	**46.82**	0.4197	**0.8723**
DXECPPI [41]	30.90	25.83	17.28	0.0825	0.5806
PPIevo [42]	78.04	25.82	27.73	0.2082	0.7329
LocFuse [43]	80.66	80.50	27.65	0.2026	0.7087
CRS [10]	91.54	96.72	36.83	0.3633	0.8196
SPAR [10]	92.09	97.40	41.13	0.3836	0.8229
**Proposed method**	**93.12**	**99.57**	39.10	**0.4421**	0.8524
